# Role of Colchicine Treatment in Tumor Necrosis Factor Receptor Associated Periodic Syndrome (TRAPS): Real-Life Data from the AIDA Network

**DOI:** 10.1155/2020/1936960

**Published:** 2020-05-27

**Authors:** Antonio Vitale, Jurgen Sota, Laura Obici, Nicola Ricco, Maria Cristina Maggio, Marco Cattalini, Piero Ruscitti, Francesco Caso, Raffaele Manna, Ombretta Viapiana, Valeria Caggiano, Giacomo Emmi, Antonella Insalaco, Davide Montin, Francesco Licciardi, Alessandra Soriano, Lorenzo Dagna, Carlo Salvarani, Vittoria Lamacchia, José Hernández-Rodríguez, Roberto Giacomelli, Bruno Frediani, Alessandra Renieri, Luca Cantarini

**Affiliations:** ^1^Research Center of Systemic Autoinflammatory Diseases and Behçet's Disease and Rheumatology-Ophthalmology Collaborative Uveitis Center, Department of Medical Sciences, Surgery and Neurosciences, University of Siena, Italy; ^2^Amyloidosis Research and Treatment Center, Fondazione IRCCS Policlinico San Matteo, Pavia, Italy; ^3^Department of Health Promotion Sciences Maternal and Infantile Care, Internal Medicine and Medical Specialties “G. D'Alessandro”, University of Palermo, Palermo, Italy; ^4^Pediatric Clinic, University of Brescia and Spedali Civili di Brescia, Brescia, Italy; ^5^Division of Rheumatology, Department of Biotechnological and Applied Clinical Science, University of L'Aquila, L'Aquila, Italy; ^6^Rheumatology Unit, Department of Clinical Medicine and Surgery, School of Medicine, University Federico II of Naples, Naples, Italy; ^7^Institute of Internal Medicine, Fondazione Policlinico Universitario A. Gemelli IRCCS, Rome, Italy; ^8^Rheumatology Section, Department of Medicine, University of Verona, Verona, Italy; ^9^Department of Experimental and Clinical Medicine, University of Florence, Florence, Italy; ^10^Division of Rheumatology, Department of Pediatric Medicine, Bambino Gesù Children's Hospital IRCCS, Rome, Italy; ^11^Division of Pediatric Immunology and Rheumatology, Department of Public Health and Pediatrics, “Regina Margherita” Children Hospital, University of Turin, Turin, Italy; ^12^Azienda Unità Sanitaria Locale-IRCCS di Reggio Emilia, Reggio Emilia, Italy; ^13^Unit of Immunology, Rheumatology, Allergy and Rare Diseases, IRCCS San Raffaele Hospital, Milan, Italy; ^14^Medical Genetics, University of Siena, Siena, Italy; ^15^Genetica Medica, Azienda Ospedaliera Universitaria Senese, Siena, Italy; ^16^Vasculitis Research Unit and Autoinflammatory Diseases Clinical Unit, Department of Autoimmune Diseases, Hospital Clinic of Barcelona, IDIBAPS, University of Barcelona, Barcelona, Spain

## Abstract

**Objective:**

To analyze the potential role of colchicine monotherapy in patients with tumor necrosis factor receptor associated periodic syndrome (TRAPS) in terms of control of clinical and laboratory manifestations.

**Methods:**

Patients with TRAPS treated with colchicine monotherapy were retrospectively enrolled; demographic, clinical and therapeutic data were collected and statistically analysed after having clustered patients according to different times at disease onset, penetrance of mutations, dosage of colchicine, and different disease manifestations.

**Results:**

24 patients (14 males; 15 with pediatric disease onset) treated with colchicine monotherapy were enrolled. Colchicine resulted in a complete response in 3 (12.5%) cases, partial response in 14 (58.3%) patients, and lack of response in 7 (29.2%) patients. There were not significant differences in colchicine response between pediatric and adult disease onset (*p* = 0.42), between low- and high-penetrance mutations (*p* = 0.62), and according to different dosages (*p* = 0.66). No significant differences were identified in the frequency of specific disease manifestations between patients experiencing any response to colchicine and patients with lack of response.

**Conclusions:**

Colchicine monotherapy is useful in a low percentage of TRAPS patients; nevertheless, it could be attempted in patients with milder phenotypes and at a lower risk of developing reactive amyloidosis.

## 1. Introduction

Tumor necrosis factor receptor associated periodic syndrome (TRAPS) is an autoinflammatory autosomal dominant disease caused by mutations in the *TNFRSF1A* gene and is characterized by typically prolonged recurrent fever attacks. Erythematous skin rash, ocular and periocular manifestations, joint involvement, and myalgia sustained by monocytic fasciitis are additional and frequent symptoms observed during flares [1]. TRAPS is characterized by a protean spectrum of clinical features and severity depending on specific gene mutations: high-penetrance mutations generally manifest with an early onset, along with severe and typical manifestations; conversely, low-penetrance mutations are more frequently identified in adult-onset patients and often lead to less severe or atypical disease features with a very low risk for amyloidosis [2–4].

Nowadays, therapy with interleukin- (IL-) 1 inhibitors is considered the standard of therapy with the highest ratio between clinical efficacy and safety profile [5, 6]. On the other hand, colchicine, which represents the gold standard treatment in patients with familial Mediterranean fever (FMF) for controlling clinical manifestations and reactive amyloidosis [7], is generally considered useless for the management of TRAPS patients [8]. Nevertheless, cases at least partially responsive to colchicine have also been described [8–10]. For this reason, we have conducted the present study to better investigate the role of colchicine as possible treatment option in TRAPS.

## 2. Methods

TRAPS patients treated with colchicine monotherapy were retrospectively enrolled in eleven Italian referral Centres. Diagnosis of TRAPS was based on suggestive clinical manifestations and supported by genetic analysis (Sanger sequencing of *TNFRSF1A* gene driven by clinical features or next-generation sequencing). In order to definitively exclude patients that could possibly benefit from colchicine administration for any other concomitant diseases, subjects fulfilling clinical diagnostic and classification criteria for Behçet's disease and periodic fever, aphthous stomatitis, pharyngitis and cervical adenitis (PFAPA) syndrome were ruled out [11–14].

The primary aim of the study was to assess clinical benefits of colchicine in TRAPS patients distinguishing cases according to different times at disease onset (pediatric- *vs* adult-onset TRAPS) and penetrance of mutations (high- *vs* low-penetrance). Secondary aims of the study were (i) to identify any difference in colchicine response on the bases of different clinical manifestations and different colchicine dosage employed and (ii) to search for any differences in colchicine role according to the response of TRAPS patients to corticosteroids, nonsteroidal anti-inflammatory drugs (NSAIDs), and biologics.

Complete response was defined as complete control of clinical and laboratory manifestations; partial response was meant as (i) a decrease in clinical severity of disease attacks after colchicine introduction testified by a mean reduction of body temperature ≥ 1°C during flares and a ≥30% decrease of erythrocyte sedimentation rate (ESR), C reactive protein (CRP), and serum amyloid A (SAA) assessed during inflammatory episodes, and (ii) a patient-reported improvement in clinical manifestations during flares for relapsing-remitting disease courses or outside of flares for chronic cases. Because of the small sample size, patients experiencing complete response and partial response were grouped together in order to compare patients presenting any colchicine response with patients undergoing failure.

Descriptive statistics was based on the evaluation of mean, standard deviation (SD), and median and interquartile range (IQR) values. For qualitative data, pairwise comparisons were performed using 2 × 2 and 2 × 3 contingency tables and applying the Fisher exact test and Freeman-Halton extension when required; Student *t*-test or Mann–Whitney *U* test, as needed, were used for pairwise comparisons of quantitative data. Normality distribution was assessed using the Shapiro-Wilk test. Correlations were performed employing Kendall's tau-b test. The SPSS software, version 24, was used for all statistical computations, always considering a significance level of 95% (*p* value < 0.05); all tests performed were two-sided.

The study has been approved by the local Ethics Committee of Azienda Ospedaliera Universitaria Senese, Siena, Italy (AIDA Project; Ref. N. 14951). The study protocol conformed to the tenets of the Declaration of Helsinki; informed consent was obtained from all patients enrolled or their legal guardians.

## 3. Results

Twenty-four TRAPS patients (14 males; 15 with pediatric disease onset) treated with colchicine during their clinical history were retrospectively enrolled. Their demographic and clinical data are summarized in Table 1. High-penetrance mutations identified in our cohort were C98Y (*n* = 2), C52Y (*n* = 1), T50M (*n* = 1), Y103_R104DEL (*n* = 1), and c.472+1G>A (*n* = 1). Low-penetrance mutations detected in the patients enrolled were R92Q (*n* = 14), D12E (*n* = 1), P46L (*n* = 1), R104Q (*n* = 1), and V95M (*n* = 1).

Colchicine resulted in a complete response in 3 (12.5%) cases, partial response in 14 (58.3%) patients, and lack of response in 7 (29.2%) patients.

**Figure 1:**
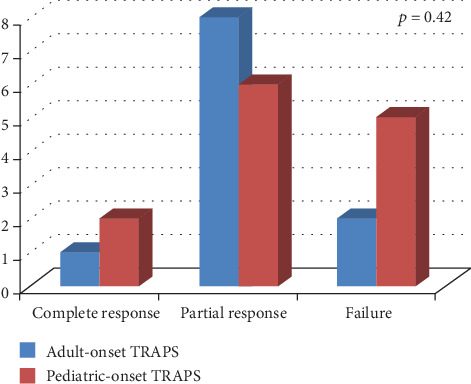
Response to colchicine treatment distinguishing patients according to different age at disease onset (pediatric- versus adult-onset).

**Figure 2:**
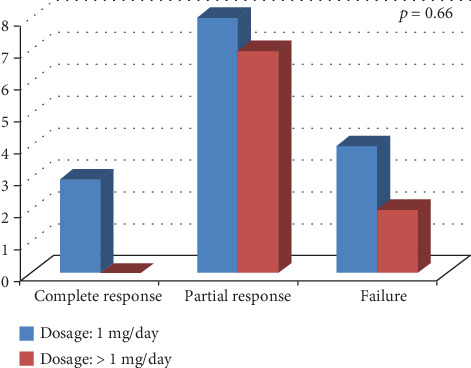
Response to colchicine administration according to different dosages employed (1 mg/day versus more than 1 mg/day).

Figure 1 shows the frequency of response to colchicine according to TRAPS age at onset. No statistically significant differences were highlighted between pediatric and adult disease onset in colchicine response (*p* = 0.42). Similarly, no significant difference was identified in colchicine response between patients treated with 1 mg/day and those treated with more than 1 mg/day (*p* = 0.66), as shown in Figure 2.

No significant differences were identified between low- and high-penetrance mutations according to the response to colchicine (*p* = 0.62). No differences were identified in the frequency of TRAPS-related clinical manifestations between patients' responsive and not responsive to colchicine, as summarized in Table 2.

Two out of three patients experiencing complete disease control did not necessitate biologic treatment; however, one patient was later treated with the IL-1*β* antagonist canakinumab because of gastrointestinal intolerance to colchicine.

Ten out of 14 patients experiencing partial efficacy were later treated with anti-IL-1 biologic agents in order to obtain complete TRAPS control; conversely, 4 patients were treated by combining colchicine with low-dose corticosteroids or NSAIDs.

**Figure 3:**
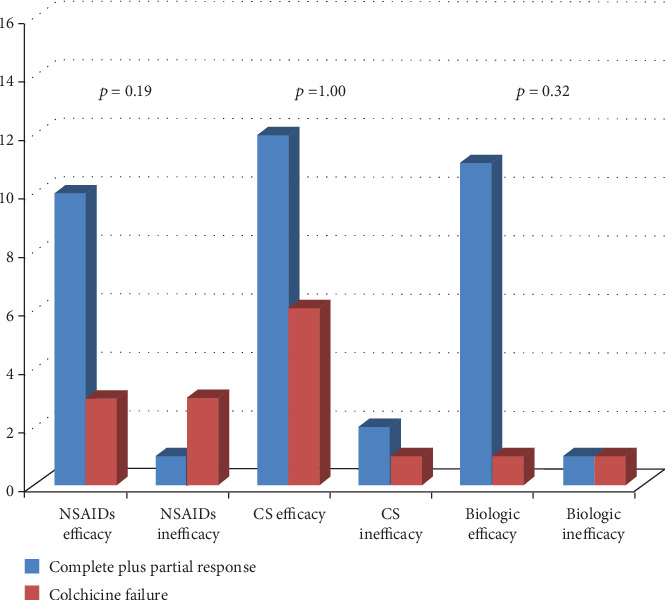
Response to colchicine administration distinguishing patients according to the final response to nonsteroidal anti-inflammatory drugs (NSAIDs), corticosteroids (CS), and biologic agents. In order to facilitate the reading of the histograms, patients experiencing complete response and partial response were meshed to be compared with patients presenting colchicine failure.

Figure 3 describes colchicine response according to the final response to biologic agents (anakinra in 9 cases, canakinumab in 4 cases, and etanercept in 1 case), NSAIDs, and corticosteroids. No differences were observed in the response to corticosteroids (*p* = 1.00), NSAIDs (*p* = 0.19), and biologic agents (*p* = 0.32) on the basis of different types of response to colchicine (failure versus complete and partial response). No significant correlation was identified between colchicine response and response to NSAIDs (*p* = 0.10), corticosteroids (*p* = 1.00), or biologics (*p* = 0.15).

Regarding laboratory inflammatory markers, acute phase reactants normalized in patients showing complete response to colchicine, while patients experiencing partial response showed a ≥30% decrease of ESR, CRP, and SAA values during attacks. However, ESR and SAA assessed during flares remained above normal values in 6/14 (42.9%) patients partially responsive to colchicine, while CRP persisted elevated in 8/14 (57.1%) patients.

## 4. Discussion

Colchicine is considered the treatment of choice in FMF patients and is used as prophylactic therapy for secondary amyloidosis in such cases [7, 15]. Indeed, colchicine has been shown to reduce the frequency of FMF attacks and prevent development of proteinuria related to amyloidosis [7]. Noteworthy, it may induce improvement of proteinuria even in patients with established amyloid nephropathy [16]. On the contrary, colchicine is commonly considered ineffective for treating patients with TRAPS, but studies specifically assessing the role of colchicine in these patients are controversial. In particular, during the early 2000s Dodé et al. and Drewe et al. reported an overall lack of response to colchicine in patients with different *TNFRSF1A* mutations [17, 18]. Conversely, basing on 25 patients treated with colchicine, Ravet et al. reported complete response in 6 cases, partial efficacy in 9 cases, and inefficacy in 10 patients [10]. Similarly, the retrospective analysis of data from the Eurofever Registry reported a beneficial effect of colchicine in 21/39 TRAPS patients, especially subjects carrying the low-penetrance R92Q mutation [8].

The results of the present study confirm the poor role of colchicine monotherapy for the management of TRAPS patients. Indeed, only three cases showed a complete response to this treatment when used before starting biologics, which currently represent the standard of treatment in such patients. Nevertheless, about half of patients enrolled in the study experienced a partial response in terms of control of clinical manifestations and decrease of systemic inflammation during flares. These results were influenced by neither the age at disease onset nor the different penetrance of mutations included in the study. In regard to this last issue, controversial results have been reported in the literature. Our findings are consistent with what is described by Ravet et al., who reported no definite difference in the efficacy of colchicine between different groups of mutations [10]. Differently, other authors have found a better response to colchicine in patients carrying low-penetrance mutation R92Q [8]. Although no statistical significances were observed, in our study, the percentage of patients benefiting from colchicine administration was even higher among patients carrying high-penetrance mutations. These discrepancies may be related to the low number of patients enrolled along with the different methodologies, especially concerning the definition of partial response. For these reasons, this matter should be further addressed in future studies conducted on a wider number of patients to better clarify whether colchicine response may be part of a genotype-phenotype correlation.

Of note, as illustrated in Figure 2, patients treated with a higher than 1 mg/day dose of colchicine did not experience a better clinical response compared to patients administered with 1 mg/day, thus suggesting no significant improvement in clinical response by increasing colchicine dosage.

No specific clinical manifestations showed to be significantly more frequent among patients with any response to colchicine compared with those showing absence of efficacy. This suggests the lack of a specific subset of TRAPS patients identifiable as more responsive to colchicine.

As shown in Figure 3, particularly worth considering is that colchicine response did not correlate with response to other treatment approaches. Consequently, response to colchicine appears independent of response to other therapies.

The limitations of this study include its retrospective nature and the small sample size of patients. In addition, our study does assess neither the role of colchicine combined with NSAIDs, corticosteroids, and biologic agents nor the trend of inflammatory markers outside of disease flares, which is a primary issue in the management of autoinflammatory diseases, as persistently high acute phase reactants may be linked to long-term complications including secondary amyloidosis. However, in spite of these limitations, at the best of our knowledge, this study represents the first attempt at primarily assessing the role of colchicine in TRAPS patients according to different clinical features.

In conclusion, evidences drawn from our study suggest that colchicine monotherapy may adequately control TRAPS manifestations only in a few cases; however, it may represent a feasible attempt to minimize immunosuppressive therapy in selected TRAPS patients with milder phenotypes and at a lower risk of developing reactive amyloidosis.

## Figures and Tables

**Table 1:** Demographic and clinical features of patients enrolled.

Demographic and clinical information	
Age at disease onset in years, mean (SD)	16.4 ± 13.1
Age at diagnosis in years, mean (SD)	30.0 ± 14.1
Age at enrollment in years, mean (SD)	35.3 ± 18.8
Male/female patients	10/14
Patients with pediatric onset-TRAPS, *n* (%)	13 (54.2)
Patients with adult onset-TRAPS, *n* (%)	11 (45.8)
High-/low-penetrance mutations	6/18
Family members with symptoms, *n* (%)	10 (41.7)
Relapsing-remitting disease course, *n* (%)	18 (75)
Chronic disease course, *n* (%)	6 (25)
Duration of flares	10.8 ± 7.9
Flares/year	2.43 ± 0.8
Amyloidosis at diagnosis	1 (4.2)
Clinical manifestations during flares, *n* (%)	
Thoracic pain	10 (41.7)
Pericarditis	9 (37.5)
Pleuritis	2 (8.3)
Abdominal pain	13 (54.2)
Pharyngitis	9 (37.5)
Oral aphthosis	6 (25)
Skin rash	9 (37.5)
Lymphadenopathy	8 (33.3)
Myalgia	15 (62.5)
Arthralgia	17 (70.8)
Arthritis	5 (20.8)
Periorbital pain	4 (16.7)
Laboratory findings	
Median erythrocyte sedimentation rate, mm/1 h (IQR)	55.7 (42)
Median C-reactive protein, mg/L (IQR)	7.7 (10.5)
Median serum amyloid A, mg/L (IQR)	59.7 (63)
Proteinuria, *n* (%)	1 (4.2)

Abbreviations: IQR: interquartile range; *n*: number of patients; SD: standard deviation; TRAPS: tumor necrosis factor associated periodic syndrome.

**Table 2:** Demographic and clinical manifestations of patients distinguished on the basis of response to colchicine (complete and partial response versus unresponsive patients).

	Colchicine-responsive patients (*n* = 17)	Colchicine-unresponsive patients (*n* = 7)	*p* value
Demographic and clinical information			
Age at disease onset in years, mean (IQR)	16.7 (20)	8.2 (9.8)	0.31
Age at diagnosis in years, mean (SD)	33.9 ± 15.4	22.5 ± 10.1	0.21
Male/female patients	3/4	7/10	1.00
Patients with pediatric onset-TRAPS, *n* (%)	8 (47.1)	5 (71.4)	0.39
Patients with adult onset-TRAPS, *n* (%)	9 (52.9)	2 (28.6)	
High-/low-penetrance mutations (%/%)	5/12 (83.3/66.7)	1/6 (16.7/33.3)	0.62
Family members with symptoms, *n* (%)	7 (41.2)	3 (42.9)	1.00
Relapsing-remitting disease course, *n* (%)	13 (76.5)	5 (71.4)	1.00
Chronic disease course, *n* (%)	4 (23.5)	2 (28.6)	
Duration of flares, (IQR)	10.1 (8)	12.33 (18)	0.90
Flares/year, (IQR)	2.29 (1)	3.00 (2)	0.22
Amyloidosis at diagnosis	1 (5.9)	0 (0)	0.54
Clinical manifestations during flares, *n* (%)			
Thoracic pain	7 (41.2)	3 (42.9)	1.00
Pericarditis	7 (41.2)	2 (28.6)	0.67
Pleuritis	2 (11.8)	0 (0)	1.00
Abdominal pain	8 (47.1)	5 (71.4)	0.39
Pharyngitis	7 (41.2)	2 (28.6)	0.67
Oral aphthosis	3 (17.6)	3 (42.9)	0.31
Skin rash	7 (41.2)	2 (28.6)	0.78
Lymphadenopathy	5 (29.4)	3 (42.9)	0.65
Myalgia	10 (58.8)	5 (71.4)	0.67
Arthralgia	12 (70.6)	5 (71.4)	1.00
Arthritis	3 (17.6)	2 (28.6)	0.61
Periorbital pain	2 (11.8)	2 (28.6)	0.55
Laboratory findings			
Median erythrocyte sedimentation rate, mm/1 h (IQR)	63.3 (51)	31 (37)	0.08
Median C-reactive protein, mg/L (IQR)	8.0 (7.9)	7.1 (10.7)	0.89
Median serum amyloid A, mg/L (IQR)	60.2 (69.4)	59.4 (89.2)	0.37
Proteinuria, *n* (%)	1 (5.9)	0 (0)	0.48

Abbreviations: IQR: interquartile range; *n*: number of patients; SD: standard deviation; TRAPS: tumor necrosis factor associated periodic syndrome.

## Data Availability

Data is available upon request to the corresponding author.
